# Percutaneous Coronary Intervention in Out-of-Hospital Cardiac Arrest Related to Acute Coronary Syndrome: A Literature Review

**DOI:** 10.3390/jcm12237275

**Published:** 2023-11-24

**Authors:** Emmanuel Gall, Donia Mafi, Tahar Ghannam, Pierre Brami, Vincent Pham, Olivier Varenne, Fabien Picard

**Affiliations:** 1 Department of Cardiology, Cochin Hospital, Hôpitaux Universitaire Paris Centre, Assistance Publique—Hôpitaux de Paris, 27 Rue du Faubourg Saint-Jacques, 75014 Paris, France; emmanuel_gall@hotmail.fr (E.G.); donia.mafi@gmail.com (D.M.); ghannamtahar96@gmail.com (T.G.); pierre.brami@aphp.fr (P.B.); pham.qtv@gmail.com (V.P.); olivier.varenne@aphp.fr (O.V.); 2 Service de Cardiologie, Hôpital Lariboisière—APHP, Université Paris-Cité, Inserm UMRS 942, 75010 Paris, France

**Keywords:** out-of-hospital cardiac arrest, invasive coronary angiogram, PCI, STEMI, NSTEMI, ischemic, bleeding

## Abstract

Out-of-hospital cardiac arrest (OHCA) continues to be a major global cause of death, affecting approximately 67 to 170 per 100,000 inhabitants annually in Europe, with a persisting high rate of mortality of up to 90% in most countries. Acute coronary syndrome (ACS) represents one of the most significant cause of cardiac arrest, and therefore invasive coronary angiography (CAG) with subsequent percutaneous coronary intervention (PCI) has emerged as a fundamental component in the management of OHCA patients. Recent evidence from large randomized controlled trials (RCTs) challenges the routine use of early CAG in the larger subgroup of patients with non-ST segment elevation myocardial infarction (NSTEMI). Additionally, emerging data suggest that individuals resuscitated from OHCA related to ACS face an elevated risk of thrombotic and bleeding events. Thus, specific invasive coronary strategies and anti-thrombotic therapies tailored to this unique setting of OHCA need to be considered for optimal in-hospital management. We sought to provide an overview of the prevalence and complexity of coronary artery disease observed in this specific population, discuss the rationale and timing for CAG after return of spontaneous circulation (ROSC), summarize invasive coronary strategies, and examine recent findings on antithrombotic therapies in the setting of ACS complicated by OHCA. By synthesizing the existing knowledge, this review aims to contribute to the understanding and optimization of care for OHCA patients to improve outcomes in this challenging clinical scenario.

## 1. Introduction

Out-of-hospital cardiac arrest (OHCA) remains a leading cause of death worldwide, with an annual incidence of 67 to 170 per 100,000 inhabitants in Europe [1] Even among those with return of spontaneous circulation (ROSC), which is achieved in 40–60%, and those who are admitted to the hospital, the prognosis is poor, with a mortality rate of up to 65%. Post resuscitation cares include diverse therapies such as targeted temperature management (TTM), advanced intensive care unit (ICU) therapies for life support, and early identification and treatment of the underlying cause of cardiac arrest.

Cardiac disease is the most common cause of OHCA [2], and the most frequent underlying cause is significant coronary artery disease (CAD), documented in up to 70% of patients [3]. With this in mind, invasive coronary angiogram (CAG) with subsequent revascularization appears to be a cornerstone in the management of OHCA patients, having as primary goal, the restoration of blood flow in the culprit coronary artery, preserving myocardial function, and preventing negative consequences of myocardial ischemia such as heart failure or lethal ventricular arrhythmias. As compared with non-complicated myocardial infarction (MI), important characteristics such as complexity and CAD observed in the specific setting of OHCA related to MI, as well as specific invasive coronary treatment strategies, need to be considered for appropriate and tailored in-hospital management. Although guidelines strongly recommend early CAG after OHCA with ST-segment elevation myocardial infarction (STEMI) [4], recent data from large randomized controlled trials (RCT) argue against a routine-based early CAG strategy in the far larger subgroup of patients with non-ST segment elevation myocardial infarction (NSTEMI) [5,6,7,8,9]. Furthermore, recent data suggest that patients who are resuscitated from OHCA related to MI are at an even greater risk of bleeding and thrombosis.

The purpose of this review is therefore to describe the prevalence and complexity of CAD observed in this specific population, understand the rationale and timing of CAG after ROSC, summarize invasive coronary treatment strategies, and review recent data on antithrombotic therapies in patients treated for ACS complicated by OHCA.

## 2. Epidemiology of CAD in OHCA

CAD is the most common trigger for OHCA in the absence of an evident non-cardiac cause. Spaulding et al. reported obstructive CAD in up to 70% and acute coronary occlusion in 40% of consecutive survivors of OHCA with no obvious non-cardiac cause regardless of initial arrest rhythm or ST-segment elevation (STE) [3]. Consistently, a recent meta-analysis showed that, among patients undergoing CAG, 75% had evidence of significant stenosis in at least one coronary artery. In 30% of patients, CAD was characterized by a single-vessel disease, whereas a multivessel disease (2 or more vessels) was present in 46% [10]. However, patients with ROSC can be divided into two groups according to the post resuscitation electrocardiogram (ECG): those who have STE on the initial post resuscitation ECG and those with non ST-segment elevation (NSTE). The prevalence of CAD differs between these two categories [10]. Among OHCA patients with STE, CAG is widely performed because an acute coronary occlusion is most often the cause of the arrest, and significant CAD has been described in 70% to 95% of cases, with an acute coronary lesion in 70% to 80% of patients [11,12]. In patients with NSTE on post resuscitation ECG, the prevalence of CAD ranges between 21% and 53% and an acute coronary lesion is identified in 25% to 35% of cases [6,7,8,12].

Another category of patients is those presenting with refractory shockable rhythm without achieved ROSC. Yannopoulos et al. described the prevalence and complexity of CAD in patients experiencing refractory cardiac arrest and transported to a cardiac catheterization laboratory for extracorporeal life support and CAG. Acute thrombotic lesions were present in 64% and chronic total occlusions were described in 33% of patients. Significant CAD (>70% stenosis) was found in 84% of patients, with single-vessel disease in 30% and multivessel disease reported in up to 70% of patients. The mean SYNTAX score of 29.4 ± 13.9 reported in the study highlights the complexity and severity of the CAD. A recent meta-analysis, taking into account 128 studies, showed that refractory cardiac arrests were associated with a higher prevalence of significant stenosis of the left main compared to patients with early ROSC (17% (95% CI, 12–24%) vs. 5.7% (95% CI, 3.1–10%); *p* = 0.002), which was also most often the culprit lesion (12% (95% CI, 7.5–18%) vs. 4.5% (95% CI, 3.5–5.7%); *p* < 0.001). Significant CAD was present in 75% (95% CI, 70–80%) of refractory cardiac arrest patients, and a culprit lesion was identified in 70% (95% CI, 60–77%), with the left anterior descending artery being the most frequent [10]. These findings suggest a greater severity of CAD in this population, with a higher prevalence of multivessel disease, which could partially explain the refractory presentation of the cardiac arrest [13,14].

The prevalence of CAD among patients with OHCA in non-shockable rhythm has been less investigated. Indeed, patients often die at the scene of the arrest despite advanced resuscitation maneuvers delivered by emergency medical services, and these patients are consequently not transported to a catheterization laboratory. Moreover, in survivors of OHCA with non-shockable rhythm, CAG is not necessarily performed, which potentially leads to ascertainment bias regarding the prevalence of CAD. However, a low prevalence of culprit lesions (1%) in consecutive patients with OHCA and non-shockable initial rhythm compared with shockable rhythm (22%) (*p* < 0.001) was reported [15].

Angiographic findings derived from most contemporary RCTs with an interest in the timing of performing CAG among patients without STE are summarized in Table 1 [5,6,7,8,9]. CAG was performed in 55% to 81.2% of cases. The presence of CAD and its severity (1, 2, or 3 vessels) are displayed below. These data reveal a substantial prevalence of CAD among patients resuscitated from OHCA with NSTEMI, with reported rates ranging from 51% to 69.9%. However, the presented data highlight the heterogenous proportion of identified culprit lesions even though the baseline characteristics and inclusion criteria were relatively similar between these five randomized controlled trials. Indeed, the proportion of culprit lesions varied from 19.9% to 45.5% in patients undergoing CAG. These heterogenous findings raise concerns about better standardization of the definition of “culprit lesion” and the need to carry out better detection of causal lesions with the potential support of intracoronary imaging [16,17].

## 3. Rationale and Timing for CAG after OHCA

### 3.1. Current Evidence

Despite progress in the field of prehospital resuscitation with the well-known “chain of survival”, more than half of patients who are initially resuscitated die in the hospital; the overall survival rate after OHCA is around 10% [18]. Recommended post resuscitation care includes TTM, advanced ICU therapies for life support, and treatment of the underlying cause of cardiac arrest.

According to series based on autopsies and immediate CAG data, acute coronary occlusion has been highlighted as the most frequent cause of OHCA, with a frequency ranging from 36% to 95% [19,20]. One of the princeps studies was published by Spaulding et al. in 1997 [3]. In this prospective monocentric study, all consecutive OHCA survivors were referred for immediate CA, and coronary lesions (acute occlusion or unstable lesion) were found in 60 of the 84 patients, including 40 acute coronary occlusions (48%) [3]. As a result of this study, the interventional cardiologist community was increasingly alerted for immediate CAG and subsequent PCI for a vast majority of OHCA regardless of ECG after cardiac arrest. Current European and American guidelines recommend immediate CAG with PCI in patients who present with STE-ACS and cardiac arrest [21,22]. However, it is still unclear whether CAG should be performed immediately at admission in all comatose patients with STE criteria on ECG regardless of neurological status on admission or well-admitted prognostic criteria such as initial rhythm, duration of no flow and low flow, and comorbidities.

**Figure 1:**
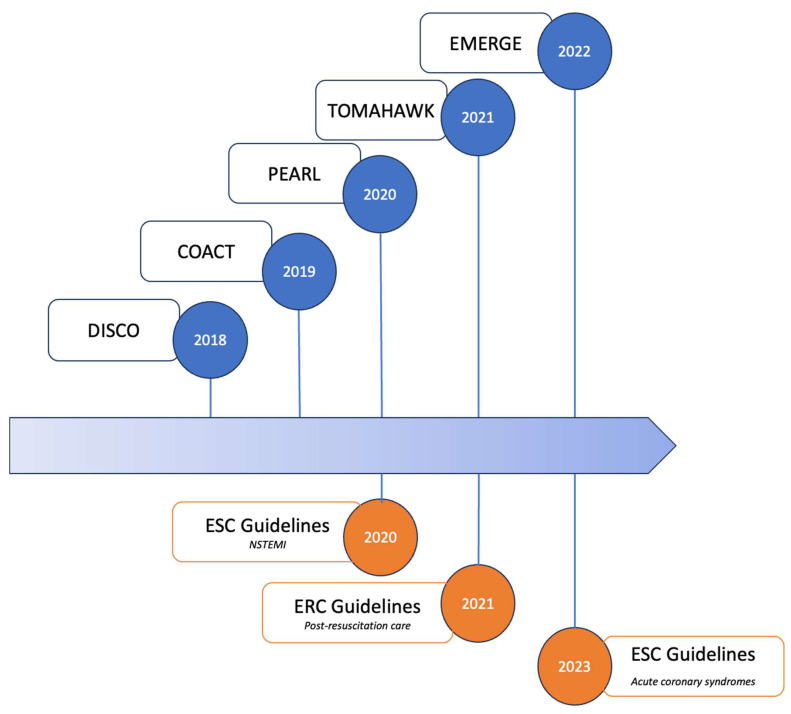
Impactful randomized clinical trials on early vs. delayed CAG in OHCA patients without ST elevation on electrocardiogram and current international guidelines. Abbreviations: COACT: coronary angiography after cardiac arrest without ST-segment elevation; DISCO: direct or subacute coronary angiography in out-of-hospital cardiac arrest; EMERGE: emergency vs. delayed coronary angiogram in survivors of out-of-hospital cardiac arrest; ERC: European Resuscitation Council; ESC: European Society of Cardiology; NSTEMI: non ST-segment elevation myocardial infarction; PEARL: randomized pilot clinical trial of early coronary angiography versus non-early coronary angiography after cardiac arrest without ST-segment elevation; TOMAHAWK: angiography after out-of-hospital cardiac arrest without ST-segment elevation.

Until recently published randomized trials [5,6,7,8,9], the role of immediate CAG in patients with NSTEMI on ECG after OHCA was a matter of debate over the past two decades (Figure 1). Indeed, data from observational studies have shown conflicting results regarding the effect of immediate CAG and subsequent PCI on outcomes in this patient group, probably due to the heterogeneity of patients experiencing OHCA without STE [23,24,25,26]. In line with the the ESC guidelines for high-risk NSTE-ACS patients until 2020, international guidelines on cardiopulmonary resuscitation recommended, an emergency CAG in selected OHCA patients after excluding obvious non-coronary causes (i.e., traumatic cardiac arrest, cerebrovascular events, respiratory failure, or intoxication) even in the absence of STE [27,28]. Rather than routine CAG after OHCA with NSTE, it has been suggested that early neurological prognostication with a dedicated score may play a role in the selection of appropriate candidates who could benefit from early CAG. Indeed, futile procedures in patients with the most severe brain damage who would die from neurological failure regardless of coronary status may be avoided using prognostic scores [29,30,31]. Moreover, unwitnessed cardiac arrest, the presence of an initial non-shockable rhythm, and prolonged duration of no/low flow should strongly argue against futile early CAG [15].

Due to these uncertainties, expert panels from international professional societies have called for additional research with randomized clinical trials in an attempt to evaluate the role of immediate CAG compared to delayed CAG with respect to survival [32,33].

The first study was the Coronary Angiography After Cardiac Arrest Without ST-Segment Elevation (COACT) trial, which randomly assigned 552 patients with OHCA without signs of STE to undergo immediate or delayed CAG after neurologic recovery [6]. All patients underwent PCI if indicated. The primary end point was survival at 90 days. At the end of follow-up, 64.5% in the immediate CAG group and 67.2% in the delayed CAG group were alive (OR = 0.89; 95% CI, 0.62 to 1.27; *p* = 0.51), with no significant difference in the median time to target temperature. However, PCI was performed in 35.2% of the patients who underwent CAG, although unstable lesions or acute occlusions were detected in only 19.9%, thus suggesting unnecessary revascularizations. Based on these results, several international guidelines have deemphasized the role of a strategy of early CAG after OHCA in patients without STE after ROSC [34,35,36]. The latest European guidelines on ACS highlights that routine immediate CAG after resuscitated CA is not recommended in hemodynamically stable patients without persistent ST-segment elevation (class III, level of evidence A) [4].

In the underpowered pilot RCT Early Coronary Angiography Versus No Early Coronary Angiography After Cardiac Arrest Without ST-Segment Elevation (PEARL) study, which was prematurely stopped, 99 patients were enrolled and the primary end point of efficacy and safety (composite of efficacy and safety measures, including efficacy measures of survival to discharge, favorable neurological status at discharge with cerebral performance category score < 2, echocardiographic measures of left ventricular ejection fraction > 50%, and a normal regional wall motion score of 16 within 24 h of admission) was not different between the two groups (55.1% versus 46.0%; *p* = 0.64) [7]. Early CAG was not associated with any significant increase in survival (55.1% versus 48.0%; *p* = 0.55). Immediate CAG revealed a potential culprit lesion for cardiac arrest in 47% of patients. A total of 14% of patients in the early-CAG group had an acute coronary occlusion. The primary end point, combining efficacy and safety, did not differ between the two groups of patients (55% vs. 46%; *p* = 0.64) [7].

Although the COACT study included only patients with a shockable rhythm, evidence regarding the general indication and timing of CAG in patients with OHCA, including those with non-shockable rhythm, was still limited until the results of the TOMAHAWK trial [8]. In this well-conducted multicenter randomized trial, a total of 554 patients with no evidence of STE and successfully resuscitated OHCA were randomly assigned to undergo either immediate or delayed CAG. The primary end point was total 30-day all-cause mortality. Secondary end points were the composite of all-cause mortality and severe neurological deficit at 30 days. At the end of follow-up, 54.0% vs. 46.0% of patients had died in the immediate angiography and delayed-angiography groups, respectively (HR, 1.28; 95% CI, 1.00 to 1.63; *p* = 0.06), with no difference in the prespecified subgroups, including the shockable vs. non-shockable rhythm subgroups. It should be noted that the proportion of acute culprit lesions (38.1% in the immediate angiography group and 43.0% in the delayed or selective angiography group) in this trial was surprisingly close to that of older studies focusing their efforts on OHCA patients with initial shockable rhythm [3,6]. Indeed, it might be interesting to know which types of lesions were considered “acute culprit lesions” and were treated. Some might have been treated without any expected benefit on mortality but with unnecessary inherent risk of complications such as thrombosis or bleeding events following percutaneous angioplasty, especially in the setting of OHCA [37]. In addition, femoral access was used in 72.0% of cases, which could also have led to increased hemorrhagic complications. However, we should acknowledge that no differences in bleeding rates were noted between the two groups [8].

Last, the Emergency vs. Delayed Coronary Angiogram In Survivors of Out-of-Hospital Cardiac Arrest (EMERGE) trial randomized 279 adult survivors from OHCA without STE on ECG to either emergency or delayed CAG [9]. The primary outcome was a 180-day survival rate with CPC 2 or less. The mean time between randomization and CAG was 0.6 h in the emergency CAG group and 55.1 h in the delayed CAG group. The 180-day survival rate with good neurological outcome was 34.1% in the emergency CAG group and 30.7% in the delayed CAG group (HR, 0.87; 95% CI, 0.65–1.15; *p* = 0.32). There was also no difference in any secondary outcome, including overall survival rate at 180 days, occurrence of shock or malignant arrhythmia within 48 h, level of myocardial dysfunction, neurological recovery at ICU discharge, and length of hospital stay. However, even though the results of this trial are in line with previous reports, it should be acknowledged that such a neutral result could be the result of the extreme underpower of the study to adequately assess the end points. Although only one-quarter of the planned number of patients was enrolled, a 4.4% absolute difference in the primary end point was observed (instead of a foreseen 10.0% absolute difference to reach a statistical difference between the two groups), thus suggesting that the results might have been totally different if the population enrollment target had been achieved.

In line with these RCTs, meta-analysis of RCTs demonstrated that, compared with delayed or no CAG, early CAG probably has no effect on mortality and may have no effect on survival with good neurological outcome [38,39]. Overall, current evidence does not support an early-CAG strategy vs. a delayed or selective strategy in hemodynamically stable comatose OHCA patients without persistent STE [4].

### 3.2. Current Guidelines: Early vs. Delayed Percutaneous Coronary Intervention

Current guidelines recommend performing an emergent CAG in comatose patients resuscitated from OHCA with STE on the post-ROSC ECG (class I, level of evidence B). Routine early CAG after resuscitated CA is not recommended in hemodynamically stable patients without persistent STE (or equivalent) (class III, level of evidence A) [4]. However, recent observational data suggest that the frequency of acute coronary occlusion in patients with OHCA and non STE did no differ between stable and unstable patients, suggesting a critical need to improve the individual risk prediction of acute coronary occlusion beyond traditional risk factors (e.g., hemodynamic status following resuscitation) [40].

## 4. PCI vs. Surgical Approach with Coronary Artery Bypass Grafting

There is a lack of studies addressing different revascularization strategies in this higher-risk population of patients with resuscitated OHCA. If there is clear acute culprit lesion, primary PCI is recommended. Performing immediate coronary artery bypass grafting (CABG) is a realistic option exclusively for conscious survivors of OHCA upon hospital arrival, specifically those with severe multivessel disease and ongoing ischemia. It is not recommended for comatose patients due to the challenge in predicting their ultimate neurological outcome.

Cardiac arrest can potentially stem from significant CAD, with a stable appearance on CAG, though the cause-and-effect connection is notably less assured. In conscious survivors of cardiac arrest, revascularization with PCI or CABG is recommended based on the extent and the severity of CAD, which can be evaluated using the Syntax score [41]. However, the decision-making process becomes intricate for comatose patients with uncertain neurological outcomes. We propose that immediate PCI for visibly obstructive stable lesions should only be conducted in patients who are hemodynamically unstable. Conversely, if a comatose survivor remains hemodynamically stable, the choice of revascularization, either with PCI or CABG, should be delayed and considered if the patient survives with minimal or no neurological issues.

## 5. PCI Strategy in OHCA Related to MI

The GRACE (Global Registry of Acute Coronary Events) registry, a large, clinical practice-based, multinational registry (n = 24,045), reported an overall incidence of major bleeding of 3.9% in patients presenting with ACS. A substantial proportion (23.8%) of the bleeding occurred at the vascular access site used for the CAG procedure [42]. In patients resuscitated from OHCA, there are no randomized data comparing the radial and femoral access for CAG procedures. The data are an extrapolation of ACS studies, and some observational studies have suggested that radial access reduced mortality in the subgroup of patients with STEMI and without OHCA [43,44]. The RIVAL trial, a large randomized multicenter trial, aimed to assess whether radial access was superior to femoral access in patients with ACS who were undergoing CAG [45]. A total of 7021 patients were randomly assigned to radial or femoral access. The primary outcome was a composite of death, MI, stroke, or non-coronary artery bypass graft-related major bleeding at 30 days. The results showed a reduction in major vascular complications in the radial group compared to the femoral group but without any difference on the primary outcome. A sub-analysis of the MATRIX trial, including 934 patients with advanced Killip class and/or cardiac arrest randomized to radial or femoral access and to bivalirudin or unfractionated heparin (UFH), demonstrated an even greater benefit from radial access and bivalirudin than in the rest of the MATRIX cohort [46]. These results contrast with the SAFARI STEMI trial, which found no differences for survival or other clinical end points at 30 days after the use of radial access vs. femoral access in patients with STEMI referred for primary PCI [47]. Given the higher risk of bleeding in the setting of OHCA related to ACS, vascular access through the radial artery might result in less bleeding than access via the femoral artery.

However, OHCA patients frequently have low radial pulse that increases the difficulty of transradial access. Femoral access is still performed in this specific setting, ranging from 4.0% to 72.0% in recent large RCTs, with the risk of major bleeding events and 30-day mortality rate [48]. Yet, if the femoral approach is mandatory, whether echo-guided puncture can reduce the risk of bleeding is still a matter of debate. A meta-analysis of five randomized trials showed that ultrasound guidance for femoral artery access in CAG allows the rate of bleeding events to decrease (OR = 0.41; 95% CI 0.20–0.83; *p* = 0.01) and puncture attempts to decrease (OR = 0.24; 95% CI: 0.19–0.31; *p* < 0.0001) [49]. More recently, the UNIVERSAL (Routine Ultrasound Guidance for Vascular Access for Cardiac Procedures) RCT, a multicenter, prospective, open-label trial, was designed to determine whether ultrasound guidance for femoral arterial access in patients undergoing CAG reduces bleeding or vascular complications in a stable population (patients with STE were excluded). A total of 621 patients were randomly assigned to the ultrasound group or to the group without any guidance. The results showed that use of ultrasound guidance for femoral access did not reduce bleeding (OR = 0.93; 95% CI: 0.55–1.56; *p* =  0.78) or vascular complications (OR = 0.67; 95% CI: 0.37–1.20; *p*  =  0.18). However, ultrasound guidance did reduce the risk of venipuncture (OR 0.24; 95% CI: 0.12–0.50; *p*  <  0.001) and the number of attempts (mean difference, −0.26; 95% CI: −0.37 to −0.16; *p*  <  0.001) with similar time to access (mean difference, −15.1; 95% CI: −45.9 to 15.8; *p* =  0.34) [50]. These results on the primary outcome must, however, be weighed against the positive results by the metanalaysis, which was included with the results from the UNIVERSAL trial, showing that ultrasound guidance was associated with reduced primary outcome of major bleeding or major vascular complications. Moreover, in a prespecified subgroup analysis performed in patients receiving vascular closure devices, there was less incidence of major bleeding and major vascular complications in the group with ultrasonography guidance. These data emphasize the fact that ultrasound guidance reduces the potential for multiple punctures, which is likely important when using a vascular closure device that will only close one site of puncture [51]. Gathering these data, a radial approach—a superficial and easily compressible site—as the default strategy should be the preferred vascular access whenever possible, including in patients with cardiogenic shock or after cardiac arrest. Ultrasonography-guided femoral access with the use of a vascular closure device should be considered as an alternative when radial access is not possible.

### 5.1. Culprit vs. Multivessel PCI

Patients presenting with acute myocardial infarction complicated by cardiac arrest often have multivessel disease [52], but revascularization strategies regarding this specific subset of patients are not well established. Among hemodynamically stable patients with STEMI and multivessel coronary artery disease, immediate multivessel PCI is noninferior to staged multivessel PCI with respect to the risk of death from any cause, nonfatal myocardial infarction, stroke, unplanned ischemia-driven revascularization, or hospitalization for heart failure at 1 year [53]. However, in the most severe conditions, such as cardiogenic shock, whether PCI should be performed immediately for stenoses in non-culprit arteries was controversial until the results of the CULPRIT SHOCK trial [54]. In this multicenter trial, 706 patients with multivessel disease, acute MI, and cardiogenic shock were randomly assigned to different revascularization strategies: either PCI of the culprit lesion only (with the option of staged revascularization of non-culprit lesions) or immediate multivessel PCI. This study included 366 patients (51.8% of the cohort) resuscitated from cardiac arrest before randomization, but patients with resuscitation longer than 30 min were excluded. Among patients who had multivessel CAD and acute MI with cardiogenic shock, the 30-day risk of composite of death or severe renal failure leading to renal-replacement therapy was lower among those who initially underwent PCI of the culprit lesion only as compared to those who underwent immediate multivessel PCI (relative risk, 0.83; 95% confidence interval (CI), 0.71 to 0.96; *p* = 0.01). Nevertheless, recent observational study showed that, among patients with acute MI with multivessel disease complicated by advanced cardiogenic shock requiring venoarterial–extracorporeal membrane oxygenation before revascularization, immediate multivessel PCI was associated with lower incidences of 30-day mortality or renal replacement therapy and 12-month follow-up mortality compared with culprit-only PCI. These findings suggest that non-culprit lesion revascularization during primary PCI could be considered in selective scenarios of cardiogenic shock, including patients with an extremely advanced form of cardiogenic shock requiring venoarterial–extracorporeal membrane oxygenation [55].

Therefore, the current guidelines of the ESC state that emergency revascularization of culprit lesion only during primary PCI is recommended independently of the delay from symptom onset in patients with acute MI complicated by cardiogenic shock (class IB recommendation) [36]. Whether these results can be extrapolated to OHCA patients is still unclear and needs further research.

### 5.2. OHCA with Stable CAD

**Figure 2:**
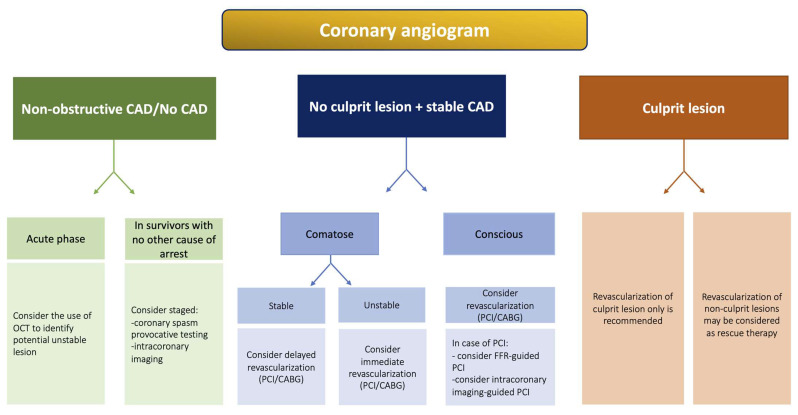
Proposed algorithm for management of patients based on angiographic findings on CAG. Abbreviations: CABG: coronary artery bypass graft; CAD: coronary artery disease; CAG: coronary angiography; CS: cardiogenic shock; FFR: fractional flow reserve; OCT: optical coherence tomography; PCI: percutaneous coronary intervention; VA-ECMO: venoarterial extracorporeal membrane oxygenation.

In patients resuscitated from OHCA without an identified culprit lesion on the coronary angiography but with significant obstructive disease with angiographically stable appearance, experts from the European Association for Percutaneous Cardiovascular Interventions (EAPCI) and Stent for Life (SFL) groups have proposed practical guidelines for revascularization even though the cause–effect relationship with cardiac arrest is unclear [32]. In comatose patients with uncertain neurological prognosis, the consensus proposes a management strategy based on hemodynamic status. In hemodynamically unstable patients, PCI of obviously obstructive “stable” lesion(s) should be performed only as a rescue therapy [56]. On the contrary, in hemodynamically stable patients, the decision for revascularization should be delayed and planned according to neurological status (i.e., no or minimal neurological sequalae). Finally, when non-obstructive CAD or no CAD has been found, a search for other causes of cardiac arrest is indicated. In survivors with favorable neurological evolution and no other obvious cause of arrest, a coronary artery spasm provocation test may be performed during a second CAG by an experienced operator using either acetylcholine or ergonovine [57]. A proposed algorithm for the management of OHCA with stable CAD is depicted in Figure 2.

### 5.3. Role of Intracoronary Imaging in OHCA Related to MI

**Figure 3:**
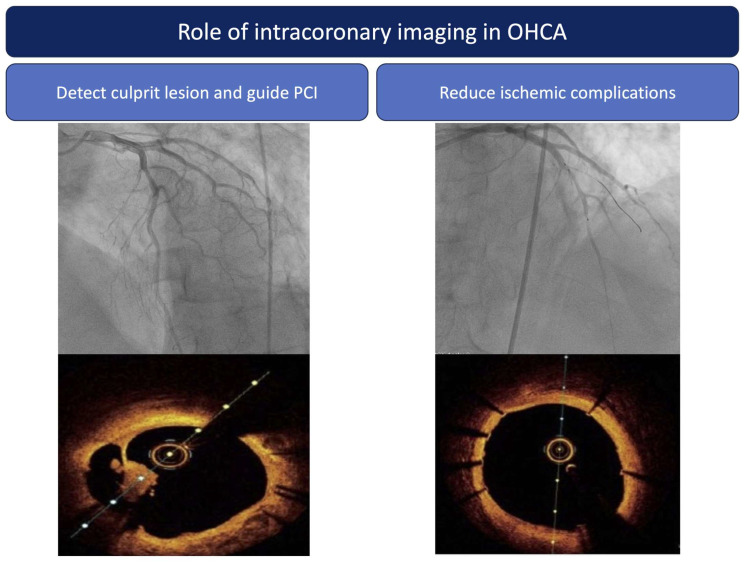
Role of intracoronary imaging in OHCA patients. Abbreviations: PCI: percutaneous coronary intervention.

Intracoronary imaging is a very useful tool for PCI in the setting of OHCA related to MI (Figure 3). Previous large observational studies have reported the safety of intra coronary imaging even considering the thrombus burden in an MI context [58]. Primarily, it can confirm the atherothrombotic or thromboembolic nature of a suspected coronary cause in OHCA survivors. Although this aspect is less of a question in OHCA patients presenting with STEMI, for whom a thrombotic occlusion can be confirmed by CAG in up to 88% of cases [25,59], the identification of the culprit lesion is more complex among those without STE on the post ROSC ECG. These findings are true for NSTEMI patients without cardiac arrest, among whom almost a third of uncertain intermediate angiographic lesions explored by OCT revealed a feature of instability (intraluminal thrombus, plaque rupture, or plaque erosion) [60]. Studies estimate that around 25 to 40% of OHCA survivors without STE have an unstable coronary lesion causative of the cardiac arrest that could be eligible for PCI [8,25]. The challenge lies in identifying such a vulnerable lesion and differentiating it from a significant stable stenosis, which would only be an incidental finding. Intracoronary imaging can provide valuable information for complete diagnostic evaluation. Therefore, it can improve patient selection compared to CAG alone and prevent unnecessary PCI with antithrombotic agents in OHCA patients who present a very high risk for stent thrombosis (5 times higher than PCI in patients without cardiac arrest) and increased hemorrhagic risk [37,61]. Regarding those uncertain situations in the absence of a clearly identified culprit lesion in the setting of an ACS, an expert consensus from EAPCI proposes to perform OCT imaging [62]. Our team performed systematic three-vessel OCT imaging in OHCA survivors with only mild atheroma based on the angiographic initial evaluation. The intracoronary imaging found at least one lesion with unstable characteristics (27% plaque rupture, 36% plaque erosion, and 59% thrombus). These findings highlight the need for a better characterization of lesions in this specific population to appropriately guide revascularization [16].

Finally, intracoronary imaging has proven its ability to guide and optimize PCI with stent implantation [63]. Indeed, it provides information regarding lesion morphology, length, diameter, and composition to achieve better lesion preparation and treatment, as well as information post PCI, such as lesion coverage, stent expansion, malaposition, and/or edge dissection, which would need further optimization to decrease the incidence of stent thrombosis, to which OHCA survivors are specifically exposed [64]. Many studies aimed to evaluate its benefits in terms of clinical events [65,66]. The recent RENOVATE-COMPLEX-PCI trial evaluated an intravascular imaging-guided PCI strategy against an angiography-guided one in 1639 patients with complex coronary lesions (50% presenting as ACS, >10% left main lesion, >20% true bifurcation lesion). In the imaging-guided group, IVUS was the most used technique (73.3%). At a median follow-up of 2.1 years, the primary end point target vessel failure (death from cardiac cause, target vessel myocardial infarction or clinically driven target vessel revascularization) occurred significantly more in the angiography-guided group than in the imaging-guided group (cumulative incidence 12.3% vs. 7.7%; *p* = 0.008). The incidence of stent thrombosis was 0.7% for the angiography-guided group and 0.1% for the imaging-guided group [67]. In line with these positive results supporting the role of intracoronary imaging to improve outcome, the ILUMIEN IV OPTIMAL PCI trial showed that OCT guidance resulted in a larger minimum stent area and could improve the rate of stent thrombosis compared with angiography-guided PCI [68]. The OCTOBER trial strengthened these results in the specific fields of complex bifurcation lesions and showed improvement in prognosis regarding MACE at 2 years compared with angiography-guided PCI [69]. All of these results strongly support the role of intracoronary imaging in complex lesions. OHCA patients with myocardial infarction, considering the complexity of an appropriate lesion diagnosis and their increased thrombotic/hemorrhagic risk, should benefit from it.

### 5.4. Balance between Ischemic and Bleeding Risk after OHCA Related to MI and Further Implications for Anti-Thrombotic Regimen

#### 5.4.1. Ischemic Risk after OHCA

In the case of ACS, administration of early and effective antithrombotic therapy is particularly important to reduce thrombotic complications and improve prognosis. Balance between thrombosis and bleeding is well recognized in patients treated for ACS, with impact on both short- and long-term prognosis [70]. Recent data suggest that patients who are resuscitated from OHCA related to MI are at even greater risk of bleeding and thrombosis [61].

Regarding thrombotic risk, several observational studies have reported higher rates of ischemic events in patients with OHCA related to MI compared with patients with ACS without OHCA, including stroke, myocardial infarction, and stent thrombosis (ST) [71]. In a recent prospective study, the incidence of ST in comatose survivors of OHCA undergoing PCI and TTM was around 19.0% despite systematic use of contemporary DES and anticoagulation/antiplatelet treatment. Moreover, ST occurred within 3 days in 62% of patients, and independent predictors of ST were longer prehospital resuscitation, lower arterial pH, and increased creatinine on admission [72]. Several hypotheses have been made to explain this ischemic risk. Although TTM may impact platelet function, with experimental studies showing conflicting effects [73,74], there is no demonstrated link to ST incidence in large clinical studies [75,76]. Second, the efficacy of oral antiplatelet therapy may be compromised in comatose patients due to delayed administration and reduced gastrointestinal motility caused by cardiac arrest and opiates [77]. However, although pharmacokinetic studies suggest an association between opiates and platelet reactivity [78], the clinical relevance of this drug–drug interaction remains controversial [79,80]. Most importantly, several studies have consistently supported the association between cardiogenic shock and ST [81,82].

#### 5.4.2. Bleeding Risk after OHCA

Patients who experience ACS complicated by OHCA are also at increased bleeding risk. Antithrombotic therapy, CPR maneuvers [83], mechanical circulatory support devices [84], and PCI, with a significant use of the femoral route, can increase bleeding risk in OHCA patients. In the COACT trial, involving 552 patients who had a cardiac arrest, transfemoral access was realized in 157/264 (59.5%) patients in the group who experienced the immediate invasive strategy of CA, compared with only 47/171 (27.5%) patients in the delayed invasive strategy group [6]. However, to perform CAG and PCI, radial access should be the preferred vascular access whenever possible, including in patients with cardiogenic shock or after cardiac arrest. Indeed, current evidence suggests that radial access is more favorable than femoral access regarding bleeding risk [48,85]. However, the presence of selection bias, where the sickest patients are more likely to undergo femoral access, complicates the interpretation of these findings.

### 5.5. Implication for Anti-Thrombotic Regimen (Figure 4)

Randomized data comparing P2Y12 receptor inhibitor or anticoagulation strategies are scarce in the setting of OHCA related to MI, and our daily practice is derived from the extrapolation of ACS trials and other pharmacodynamic or observational studies. Indeed, current guidelines on antithrombotic treatment in this particular setting is based on expert opinion [37,86]. Briefly, in patients with OHCA-related MI, 75–250 mg of intravenous aspirin may be preferable to oral aspirin loading. Regarding P2Y12 inhibitors, prasugrel and ticagrelor should be used (as opposed to clopidogrel) when there is no excessive bleeding risk. Parenteral antithrombotic therapy should be considered to cover the period before the onset of oral P2Y12 inhibitor activity. Cangrelor is preferred due to the lower bleeding risk, unless there is no reflow or bailout during PCI, when glycoprotein IIb/IIIa inhibitors can be considered. There is no evidence to recommend routine glycoprotein IIb/IIIa inhibitor use, which increases the risk of bleeding and should therefore be carefully considered in this setting except in the case of slow flow, no reflow, or high intraprocedural thrombus burden associated with low expected bleeding risk. Regarding anticoagulation treatment, there are no clinical studies assessing the benefit of heparin or the choice of anticoagulant on outcomes in patients with OHCA. No specific recommendation regarding the use of unfractionated heparin exists in this context, except for increased awareness of heparin-induced thrombocytopenia risk. Nevertheless, unfractionated heparin is the heparin of choice for cardiac arrest patients either before or during PCI or for continued anticoagulation after PCI because of its favorable pharmacodynamic profile, its reversibility mediated by protamine sulphate, its low cost, and the ability to monitor its effectiveness by measuring antiXa. In addition, with the high prevalence of acute kidney and liver injury in this population, low-molecular-weight heparin might not be the preferred heparin. It should, however, be noticed that in patients managed after cardiac arrest, there has been evidence of heparin-impaired clearance secondary to TTM, leading to drastically reduced heparin requirements in patients under TTM.

**Figure 4:**
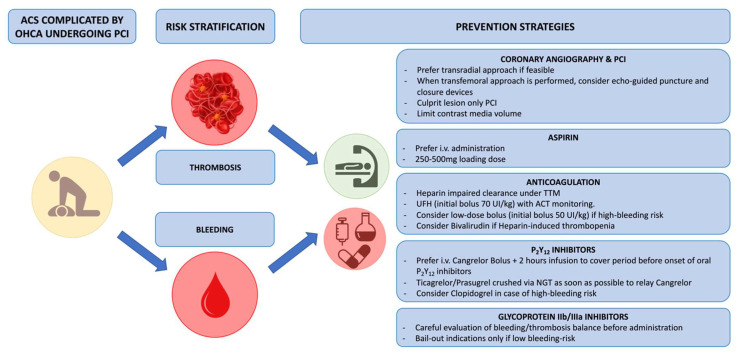
Balance between ischemic and bleeding risk in OHCA patients and further implication for anti-thrombotic regimen. Abbreviations: ACS: acute coronary syndrome; ACT: activated clotting time; I.V.: intravenous; NGT: nasogastric tube; OHCA: out-of-hospital cardiac arrest; PCI: percutaneous coronary intervention; TTM: targeted temperature management; UFH: unfractionated heparin.

## 6. Conclusions

Complex but treatable CAD is prevalent in OHCA patients. Although patients with clear STE on post resuscitation ECG have not been clearly evaluated and should still benefit from early CAG, current evidence from several RCTs regarding OHCA patients without STE does not support a strategy with early CAG compared to a delayed or selective strategy. Regarding technical aspects in this specific setting, revascularization of culprit lesions only during primary PCI is recommended in patients with cardiogenic shock, whereas complete revascularization in patients without shock still warrants further research. Vascular access through the radial artery might result in less bleeding than access via the femoral artery during CAG, and ultrasonography-guided femoral access with the use of a vascular closure device should be considered as an alternative when radial access is not possible. In the absence of acute coronary occlusion, intracoronary imaging can be helpful to detect plaque rupture, erosion, and intracoronary thrombus but could also lead to better stent implantation and help to reduce the risk of stent thrombosis, which is particularly important in this context. Importantly, more reliable information is needed to optimize antithrombotic therapy in these very-high-risk patients, in whom minimizing the risk of thrombosis and bleeding is critical to improve outcomes.

## Figures and Tables

**Table 1:** Prevalence, extent, and severity of CAD and subsequent revascularization in patients with OHCA and without ST-segment elevation: insight from recent RCTs.

Author, Year of Publication	Period of Inclusion	N	Performed CAG (%)	CAD Burden (%)					Culprit Lesion (%)	Chronic Total Occlusion (%)	Revascularization (%)	
				Normal or Insignifiant Disease	Presence of Obstructive Stable CAD	1 Vessel	2 Vessels	3 Vessels			PCI Performed among Patients Undergoing CAG	CABG
Elfwen et al., 2018 [5]	2015–2017	79	55	NC	NC	NC	NC	NC	19.9	5.3	39.5	NC
Lemkes et al., 2019 [6]	2015–2018	552	81.2	35	64.9	27.7	20.4	17	5.0	36.8	35.2	6.9
Kern et al., 2020 [7]	2015–2018	99	73.7	30.1	69.9	30.1	17.8	13.7	45.2	15.1	20.6	4.1
Desch et al., 2021 [8]	2016–2019	554	78.9	34.7	65.2	13.9	13.9	37.4	40.0	NC	39.6	NC
Berlemont et al., 2022 [9]	2017–2020	279	71.7	49	51	16.5	18	16.5	NC	NC	27.5	NC

Abbreviations: RCT: randomized control trial; CAG: coronary angiography; CAD: coronary artery disease; PCI: percutaneous coronary intervention; CABG: coronary artery bypass graft.

## Abbreviations

ACS	acute coronary syndrome
CAD	coronary artery disease
ECG	electrocardiogram
ESC	European Society of Cardiology
CAG	coronary angiography
ICU	intensive care units
MI	myocardial infarction
NSTE	non-ST-segment elevation
NSTEMI	non-ST segment elevation myocardial infarction
OHCA	out-of-hospital cardiac arrest
PCI	percutaneous coronary intervention
RCT	randomized controlled trial
ROSC	return of spontaneous circulation
STE	ST-segment elevation
STEMI	ST-segment elevation myocardial infarction
TTM	targeted temperature management

## References

[1] Gräsner J.-T., Herlitz J., Tjelmeland I.B.M., Wnent J., Masterson S., Lilja G., Bein B., Böttiger B.W., Rosell-Ortiz F., Nolan J.P. (2021). European Resuscitation Council Guidelines 2021: Epidemiology of cardiac arrest in Europe. Resuscitation.

[2] Engdahl J., Holmberg M., Karlson B.W., Luepker R., Herlitz J. (2002). The epidemiology of out-of-hospital “sudden” cardiac arrest. Resuscitation.

[3] Spaulding C.M., Joly L.M., Rosenberg A., Monchi M., Weber S.N., Dhainaut J.F., Carli P. (1997). Immediate coronary angiography in survivors of out-of-hospital cardiac arrest. N. Engl. J. Med..

[4] Byrne R.A., Rossello X., Coughlan J.J., Barbato E., Berry C., Chieffo A., Claeys M.J., Dan G.-A., Dweck M.R., Galbraith M. (2023). 2023 ESC Guidelines for the management of acute coronary syndromes. Eur. Heart J..

[5] Elfwén L., Lagedal R., Nordberg P., James S., Oldgren J., Böhm F., Lundgren P., Rylander C., van der Linden J., Hollenberg J. (2019). Direct or subacute coronary angiography in out-of-hospital cardiac arrest (DISCO)-An initial pilot-study of a randomized clinical trial. Resuscitation.

[6] Lemkes J.S., Janssens G.N., van der Hoeven N.W., Jewbali L.S.D., Dubois E.A., Meuwissen M., Rijpstra T.A., Bosker H.A., Blans M.J., Bleeker G.B. (2019). Coronary Angiography after Cardiac Arrest without ST-Segment Elevation. N. Engl. J. Med..

[7] Kern K.B., Radsel P., Jentzer J.C., Seder D.B., Lee K.S., Lotun K., Janardhanan R., Stub D., Hsu C.-H., Noc M. (2020). Randomized Pilot Clinical Trial of Early Coronary Angiography versus No Early Coronary Angiography after Cardiac Arrest without ST-Segment Elevation: The PEARL Study. Circulation.

[8] Desch S., Freund A., Akin I., Behnes M., Preusch M.R., Zelniker T.A., Skurk C., Landmesser U., Graf T., Eitel I. (2021). Angiography after Out-of-Hospital Cardiac Arrest without ST-Segment Elevation. N. Engl. J. Med..

[9] Hauw-Berlemont C., Lamhaut L., Diehl J.-L., Andreotti C., Varenne O., Leroux P., Lascarrou J.-B., Guerin P., Loeb T., Roupie E. (2022). Emergency vs Delayed Coronary Angiogram in Survivors of Out-of-Hospital Cardiac Arrest: Results of the Randomized, Multicentric EMERGE Trial. JAMA Cardiol..

[10] Scquizzato T., Sofia R., Gazzato A., Sudano A., Altizio S., Biondi-Zoccai G., Ajello S., Scandroglio A.M., Landoni G., Zangrillo A. (2023). Coronary angiography findings in resuscitated and refractory out-of-hospital cardiac arrest: A systematic review and meta-analysis. Resuscitation.

[11] Garcia S., Drexel T., Bekwelem W., Raveendran G., Caldwell E., Hodgson L., Wang Q., Adabag S., Mahoney B., Frascone R. (2016). Early Access to the Cardiac Catheterization Laboratory for Patients Resuscitated From Cardiac Arrest Due to a Shockable Rhythm: The Minnesota Resuscitation Consortium Twin Cities Unified Protocol. J. Am. Heart Assoc..

[12] Kern K.B., Lotun K., Patel N., Mooney M.R., Hollenbeck R.D., McPherson J.A., McMullan P.W., Unger B., Hsu C.-H., Seder D.B. (2015). Outcomes of Comatose Cardiac Arrest Survivors with and without ST-Segment Elevation Myocardial Infarction: Importance of Coronary Angiography. JACC Cardiovasc. Interv..

[13] Yannopoulos D., Bartos J.A., Raveendran G., Conterato M., Frascone R.J., Trembley A., John R., Connett J., Benditt D.G., Lurie K.G. (2017). Coronary Artery Disease in Patients With Out-of-Hospital Refractory Ventricular Fibrillation Cardiac Arrest. J. Am. Coll. Cardiol..

[14] Lamhaut L., Tea V., Raphalen J.-H., An K., Dagron C., Jouffroy R., Jouven X., Cariou A., Baud F., Spaulding C. (2018). Coronary lesions in refractory out of hospital cardiac arrest (OHCA) treated by extra corporeal pulmonary resuscitation (ECPR). Resuscitation.

[15] Voicu S., Bajoras V., Gall E., Deye N., Malissin I., Dillinger J.-G., Benajiba C., Logeart D., Henry P., Megarbane B. (2020). Immediate coronary angiogram in out-of-hospital cardiac arrest patients with non-shockable initial rhythm and without ST-segment elevation—Is there a clinical benefit?. Resuscitation.

[16] Degrell P., Picard F., Combaret N., Mogi S., Motreff P., Cariou A., Varenne O. (2019). Coronary atherothrombosis in cardiac arrest survivors without ST-segment elevation on ECG. Resuscitation.

[17] Degrell P., Varenne O. (2019). Coronary Angiography after Cardiac Arrest without ST-Segment Elevation. N. Engl. J. Med..

[18] Tsao C.W., Aday A.W., Almarzooq Z.I., Anderson C.A.M., Arora P., Avery C.L., Baker-Smith C.M., Beaton A.Z., Boehme A.K., Buxton A.E. (2023). Heart Disease and Stroke Statistics-2023 Update: A Report From the American Heart Association. Circulation.

[19] Davies M.J., Thomas A. (1984). Thrombosis and acute coronary-artery lesions in sudden cardiac ischemic death. N. Engl. J. Med..

[20] Myerburg R.J., Conde C.A., Sung R.J., Mayorga-Cortes A., Mallon S.M., Sheps D.S., Appel R.A., Castellanos A. (1980). Clinical, electrophysiologic and hemodynamic profile of patients resuscitated from prehospital cardiac arrest. Am. J. Med..

[21] Ibanez B., James S., Agewall S., Antunes M.J., Bucciarelli-Ducci C., Bueno H., Caforio A.L.P., Crea F., Goudevenos J.A., Halvorsen S. (2018). 2017 ESC Guidelines for the management of acute myocardial infarction in patients presenting with ST-segment elevation: The Task Force for the management of acute myocardial infarction in patients presenting with ST-segment elevation of the European Society of Cardiology (ESC). Eur. Heart J..

[22] O’Gara P.T., Kushner F.G., Ascheim D.D., Casey D.E., Chung M.K., de Lemos J.A., Ettinger S.M., Fang J.C., Fesmire F.M., Franklin B.A. (2013). 2013 ACCF/AHA guideline for the management of ST-elevation myocardial infarction: A report of the American College of Cardiology Foundation/American Heart Association Task Force on Practice Guidelines. J. Am. Coll. Cardiol..

[23] Bro-Jeppesen J., Kjaergaard J., Wanscher M., Pedersen F., Holmvang L., Lippert F.K., Møller J.E., Køber L., Hassager C. (2012). Emergency coronary angiography in comatose cardiac arrest patients: Do real-life experiences support the guidelines?. Eur. Heart J. Acute Cardiovasc. Care.

[24] Dankiewicz J., Nielsen N., Annborn M., Cronberg T., Erlinge D., Gasche Y., Hassager C., Kjaergaard J., Pellis T., Friberg H. (2015). Survival in patients without acute ST elevation after cardiac arrest and association with early coronary angiography: A post hoc analysis from the TTM trial. Intensive Care Med..

[25] Dumas F., Bougouin W., Geri G., Lamhaut L., Rosencher J., Pène F., Chiche J.-D., Varenne O., Carli P., Jouven X. (2016). Emergency Percutaneous Coronary Intervention in Post-Cardiac Arrest Patients Without ST-Segment Elevation Pattern: Insights From the PROCAT II Registry. JACC Cardiovasc. Interv..

[26] Caniato F., Lazzeri C., Bonizzoli M., Mattesini A., Batacchi S., Cappelli F., Di Mario C., Peris A. (2023). Urgent coronary angiography in out-of-hospital cardiac arrest: A retrospective single centre investigation. J. Cardiovasc. Med..

[27] Nolan J.P., Soar J., Cariou A., Cronberg T., Moulaert V.R.M., Deakin C.D., Bottiger B.W., Friberg H., Sunde K., Sandroni C. (2015). European Resuscitation Council and European Society of Intensive Care Medicine 2015 guidelines for post-resuscitation care. Intensive Care Med..

[28] Welsford M., Nikolaou N.I., Beygui F., Bossaert L., Ghaemmaghami C., Nonogi H., O’Connor R.E., Pichel D.R., Scott T., Walters D.L. (2015). Part 5: Acute Coronary Syndromes: 2015 International Consensus on Cardiopulmonary Resuscitation and Emergency Cardiovascular Care Science With Treatment Recommendations. Circulation.

[29] Maupain C., Bougouin W., Lamhaut L., Deye N., Diehl J.-L., Geri G., Perier M.-C., Beganton F., Marijon E., Jouven X. (2016). The CAHP (Cardiac Arrest Hospital Prognosis) score: A tool for risk stratification after out-of-hospital cardiac arrest. Eur. Heart J..

[30] Pareek N., Beckley-Hoelscher N., Kanyal R., Cannata A., Kordis P., Sunderland N., Kirresh A., Nevett J., Fothergill R., Webb I. (2022). MIRACLE2 Score and SCAI Grade to Identify Patients With Out-of-Hospital Cardiac Arrest for Immediate Coronary Angiography. JACC Cardiovasc. Interv..

[31] Aldous R., Roy R., Cannata A., Abdrazak M., Mohanan S., Beckley-Hoelscher N., Stahl D., Kanyal R., Kordis P., Sunderland N. (2023). MIRACLE2 Score Compared with Downtime and Current Selection Criterion for Invasive Cardiovascular Therapies After OHCA. JACC Cardiovasc. Interv..

[32] Noc M., Fajadet J., Lassen J.F., Kala P., MacCarthy P., Olivecrona G.K., Windecker S., Spaulding C. (2014). Invasive coronary treatment strategies for out-of-hospital cardiac arrest: A consensus statement from the European association for percutaneous cardiovascular interventions (EAPCI)/stent for life (SFL) groups. EuroIntervention.

[33] Rab T., Kern K.B., Tamis-Holland J.E., Henry T.D., McDaniel M., Dickert N.W., Cigarroa J.E., Keadey M., Ramee S., Interventional Council, American College of Cardiology (2015). Cardiac Arrest: A Treatment Algorithm for Emergent Invasive Cardiac Procedures in the Resuscitated Comatose Patient. J. Am. Coll. Cardiol..

[34] Panchal A.R., Bartos J.A., Cabañas J.G., Donnino M.W., Drennan I.R., Hirsch K.G., Kudenchuk P.J., Kurz M.C., Lavonas E.J., Morley P.T. (2020). Part 3: Adult Basic and Advanced Life Support: 2020 American Heart Association Guidelines for Cardiopulmonary Resuscitation and Emergency Cardiovascular Care. Circulation.

[35] Nolan J.P., Sandroni C., Böttiger B.W., Cariou A., Cronberg T., Friberg H., Genbrugge C., Haywood K., Lilja G., Moulaert V.R.M. (2021). European Resuscitation Council and European Society of Intensive Care Medicine guidelines 2021: Post-resuscitation care. Intensive Care Med..

[36] Collet J.P., Thiele H., Barbato E., Barthélémy O., Bauersachs J., Bhatt D.L., Dendale P., Dorobantu M., Edvardsen T., Folliguet T. (2021). 2020 ESC Guidelines for the management of acute coronary syndromes in patients presenting without persistent ST-segment elevation. Eur. Heart J..

[37] Gall E., Lafont A., Varenne O., Dumas F., Cariou A., Picard F. (2021). Balancing thrombosis and bleeding after out-of-hospital cardiac arrest related to acute coronary syndrome: A literature review. Arch. Cardiovasc. Dis..

[38] Goel V., Bloom J.E., Dawson L., Shirwaiker A., Bernard S., Nehme Z., Donner D., Hauw-Berlemont C., Vilfaillot A., Chan W. (2023). Early versus deferred coronary angiography following cardiac arrest. A systematic review and meta-analysis. Resusc. Plus.

[39] Al Lawati K., Forestell B., Binbraik Y., Sharif S., Ainsworth C., Mathew R., Amin F., Al Fawaz M., Pinilla-Echeverri N., Belley-Côté E. (2023). Early Versus Delayed Coronary Angiography After Out-of-Hospital Cardiac Arrest Without ST-Segment Elevation-A Systematic Review and Meta-Analysis of Randomized Controlled Trials. Crit. Care Explor..

[40] Spirito A., Vaisnora L., Papadis A., Iacovelli F., Sardu C., Selberg A., Bär S., Kavaliauskaite R., Temperli F., Asatryan B. (2023). Acute Coronary Occlusion in Patients With Non-ST-Segment Elevation Out-of-Hospital Cardiac Arrest. J. Am. Coll. Cardiol..

[41] Farooq V., van Klaveren D., Steyerberg E.W., Meliga E., Vergouwe Y., Chieffo A., Kappetein A.P., Colombo A., Holmes D.R., Mack M. (2013). Anatomical and clinical characteristics to guide decision making between coronary artery bypass surgery and percutaneous coronary intervention for individual patients: Development and validation of SYNTAX score II. Lancet.

[42] Moscucci M., Fox K.A., Cannon C.P., Klein W., López-Sendón J., Montalescot G., White K., Goldberg R.J. (2003). Predictors of major bleeding in acute coronary syndromes: The Global Registry of Acute Coronary Events (GRACE). Eur. Heart J..

[43] Chase A.J., Fretz E.B., Warburton W.P., Klinke W.P., Carere R.G., Pi D., Berry B., Hilton J.D. (2008). Association of the arterial access site at angioplasty with transfusion and mortality: The M.O.R.T.A.L study (Mortality benefit Of Reduced Transfusion after percutaneous coronary intervention via the Arm or Leg). Heart Br. Card. Soc..

[44] Montalescot G., Ongen Z., Guindy R., Sousa A., Lu S.-Z., Pahlajani D., Pellois A., Vicaut E., RIVIERA Investigators (2008). Predictors of outcome in patients undergoing PCI. Results of the RIVIERA study. Int. J. Cardiol..

[45] Jolly S.S., Yusuf S., Cairns J., Niemelä K., Xavier D., Widimsky P., Budaj A., Niemelä M., Valentin V., Lewis B.S. (2011). Radial versus femoral access for coronary angiography and intervention in patients with acute coronary syndromes (RIVAL): A randomised, parallel group, multicentre trial. Lancet.

[46] Gargiulo G., Valgimigli M., Sunnåker M., Vranckx P., Frigoli E., Leonardi S., Spirito A., Gragnano F., Manavifar N., Galea R. (2020). Choice of access site and type of anticoagulant in acute coronary syndromes with advanced Killip class or out-of-hospital cardiac arrest. Rev. Espanola Cardiol. Engl. Ed..

[47] Le May M., Wells G., So D., Chong A.Y., Dick A., Froeschl M., Glover C., Hibbert B., Marquis J.-F., Blondeau M. (2020). Safety and Efficacy of Femoral Access vs Radial Access in ST-Segment Elevation Myocardial Infarction: The SAFARI-STEMI Randomized Clinical Trial. JAMA Cardiol..

[48] Guedeney P., Thiele H., Kerneis M., Barthélémy O., Baumann S., Sandri M., de Waha-Thiele S., Fuernau G., Rouanet S., Piek J.J. (2020). Radial versus femoral artery access for percutaneous coronary artery intervention in patients with acute myocardial infarction and multivessel disease complicated by cardiogenic shock: Subanalysis from the CULPRIT-SHOCK trial. Am. Heart J..

[49] Marquis-Gravel G., Tremblay-Gravel M., Lévesque J., Généreux P., Schampaert E., Palisaitis D., Doucet M., Charron T., Terriault P., Tessier P. (2018). Ultrasound guidance versus anatomical landmark approach for femoral artery access in coronary angiography: A randomized controlled trial and a meta-analysis. J. Interv. Cardiol..

[50] Jolly S.S., AlRashidi S., d’Entremont M.-A., Alansari O., Brochu B., Heenan L., Skuriat E., Tyrwhitt J., Raco M., Tsang M. (2022). Routine Ultrasonography Guidance for Femoral Vascular Access for Cardiac Procedures: The UNIVERSAL Randomized Clinical Trial. JAMA Cardiol..

[51] d’Entremont M.-A., Alrashidi S., Alansari O., Brochu B., Heenan L., Skuriat E., Tyrwhitt J., Raco M., Tsang M., Valettas N. (2023). Ultrasound-guided femoral access in patients with vascular closure devices: A prespecified analysis of the randomised UNIVERSAL trial. EuroIntervention.

[52] Sugizaki Y., Shinke T., Doi T., Igarashi N., Otake H., Kawamori H., Hirata K.-I. (2019). Impact of the angiographic burden on the incidence of out-of-hospital ventricular fibrillation in patients with acute myocardial infarction. Heart Vessel..

[53] Stähli B.E., Varbella F., Linke A., Schwarz B., Felix S.B., Seiffert M., Kesterke R., Nordbeck P., Witzenbichler B., Lang I.M. (2023). Timing of Complete Revascularization with Multivessel PCI for Myocardial Infarction. N. Engl. J. Med..

[54] Thiele H., Akin I., Sandri M., Fuernau G., de Waha S., Meyer-Saraei R., Nordbeck P., Geisler T., Landmesser U., Skurk C. (2017). PCI Strategies in Patients with Acute Myocardial Infarction and Cardiogenic Shock. N. Engl. J. Med..

[55] Choi K.H., Yang J.H., Park T.K., Lee J.M., Song Y.B., Hahn J.-Y., Choi S.-H., Ahn C.-M., Yu C.W., Park I.H. (2023). Culprit-Only versus Immediate Multivessel Percutaneous Coronary Intervention in Patients with Acute Myocardial Infarction Complicating Advanced Cardiogenic Shock Requiring Venoarterial-Extracorporeal Membrane Oxygenation. J. Am. Heart Assoc..

[56] Mylotte D., Morice M.C., Eltchaninoff H., Garot J., Louvard Y., Lefèvre T., Garot P. (2013). Primary percutaneous coronary intervention in patients with acute myocardial infarction, resuscitated cardiac arrest, and cardiogenic shock: The role of primary multivessel revascularization. JACC Cardiovasc. Interv..

[57] Montalescot G., Sechtem U., Achenbach S., Andreotti F., Arden C., Budaj A., Bugiardini R., Crea F., Cuisset T., Task Force Members (2013). 2013 ESC guidelines on the management of stable coronary artery disease: The Task Force on the management of stable coronary artery disease of the European Society of Cardiology. Eur. Heart J..

[58] Kaziród-Wolski K., Sielski J., Gąsior M., Bujak K., Hawranek M., Pyka Ł., Gierlotka M., Pawłowski T., Siudak Z. (2023). Factors Affecting Short- and Long-Term Survival of Patients with Acute Coronary Syndrome Treated Invasively Using Intravascular Ultrasound and Fractional Flow Reserve: Analysis of Data from the Polish Registry of Acute Coronary Syndromes 2017–2020. Kardiol. Pol. (Pol. Heart J.).

[59] Sideris G., Voicu S., Dillinger J.G., Stratiev V., Logeart D., Broche C., Vivien B., Brun P.-Y., Deye N., Capan D. (2011). Value of post-resuscitation electrocardiogram in the diagnosis of acute myocardial infarction in out-of-hospital cardiac arrest patients. Resuscitation.

[60] Bogale N., Lempereur M., Sheikh I., Wood D., Saw J., Fung A. (2016). Optical coherence tomography (OCT) evaluation of intermediate coronary lesions in patients with NSTEMI. Cardiovasc. Revasculariz. Med. Mol. Interv..

[61] Picard F., Llitjos J.-F., Diefenbronn M., Laghlam D., Seret G., Sokoloff A., Cariou A., Dumas F., Varenne O. (2019). The balance of thrombosis and hemorrhage in STEMI patients with or without associated cardiac arrest: An observational study. Resuscitation.

[62] Johnson T.W., Räber L., di Mario C., Bourantas C., Jia H., Mattesini A., Gonzalo N., de la Torre Hernandez J.M., Prati F., Koskinas K. (2019). Clinical use of intracoronary imaging. Part 2: Acute coronary syndromes, ambiguous coronary angiography findings, and guiding interventional decision-making: An expert consensus document of the European Association of Percutaneous Cardiovascular Interventions. Eur. Heart J..

[63] Brami P., Picard F., Seret G., Fischer Q., Pham V., Varenne O. (2023). Intracoronary imaging in addition to coronary angiography for patients with out-of-hospital cardiac arrest: More information for better care?. Arch. Cardiovasc. Dis..

[64] Souteyrand G., Arbustini E., Motreff P., Gatto L., Di Vito L., Marco V., Amabile N., Chisari A., Kodama T., Romagnoli E. (2015). Serial optical coherence tomography imaging of ACS-causing culprit plaques. EuroIntervention.

[65] Stone G.W., Witzenbichler B., Weisz G., Rinaldi M.J., Neumann F.-J., Metzger D.C., Henry T.D., Cox D.A., Duffy P.L., Mazzaferri E. (2013). Platelet reactivity and clinical outcomes after coronary artery implantation of drug-eluting stents (ADAPT-DES): A prospective multicentre registry study. Lancet.

[66] Räber L., Mintz G.S., Koskinas K.C., Johnson T.W., Holm N.R., Onuma Y., Radu M.D., Joner M., Yu B., Jia H. (2018). Clinical use of intracoronary imaging. Part 1: Guidance and optimization of coronary interventions. An expert consensus document of the European Association of Percutaneous Cardiovascular Interventions. Eur. Heart J..

[67] Lee J.M., Choi K.H., Song Y.B., Lee J.-Y., Lee S.-J., Lee S.Y., Kim S.M., Yun K.H., Cho J.Y., Kim C.J. (2023). Intravascular Imaging-Guided or Angiography-Guided Complex PCI. N. Engl. J. Med..

[68] Ali Z.A., Landmesser U., Maehara A., Matsumura M., Shlofmitz R.A., Guagliumi G., Price M.J., Hill J.M., Akasaka T., Prati F. (2023). Optical Coherence Tomography-Guided versus Angiography-Guided PCI. N. Engl. J. Med..

[69] Holm N.R., Andreasen L.N., Neghabat O., Laanmets P., Kumsars I., Bennett J., Olsen N.T., Odenstedt J., Hoffmann P., Dens J. (2023). OCT or Angiography Guidance for PCI in Complex Bifurcation Lesions. N. Engl. J. Med..

[70] Ducrocq G., Schulte P.J., Budaj A., Cornel J.H., Held C., Himmelmann A., Husted S., Storey R.F., Cannon C.P., Becker R.C. (2017). Balancing the risk of spontaneous ischemic and major bleeding events in acute coronary syndromes. Am. Heart J..

[71] Spirito A., Gargiulo G., Siontis G.C.M., Mitsis A., Billinger M., Windecker S., Valgimigli M. (2021). Cardiovascular mortality and morbidity in patients undergoing percutaneous coronary intervention after out-of-hospital cardiac arrest: A systematic review and meta-analysis. EuroIntervention.

[72] Rauber M., Nicol P., Sabic E., Joner M., Noc M. (2022). Timing and predictors of definite stent thrombosis in comatose survivors of out-of-hospital cardiac arrest undergoing percutaneous coronary intervention and therapeutic hypothermia (ST-OHCA study). EuroIntervention.

[73] Xavier R.G., White A.E., Fox S.C., Wilcox R.G., Heptinstall S. (2007). Enhanced platelet aggregation and activation under conditions of hypothermia. Thromb. Haemost..

[74] Steblovnik K., Blinc A., Bozic-Mijovski M., Kranjec I., Melkic E., Noc M. (2015). Platelet reactivity in comatose survivors of cardiac arrest undergoing percutaneous coronary intervention and hypothermia. EuroIntervention.

[75] Rosillo S.O., Lopez-de-Sa E., Iniesta A.M., de Torres F., del Prado S., Rey J.R., Armada E., Moreno R., López-Sendón J.L. (2014). Is therapeutic hypothermia a risk factor for stent thrombosis?. J. Am. Coll. Cardiol..

[76] Shah N., Chaudhary R., Mehta K., Agarwal V., Garg J., Freudenberger R., Jacobs L., Cox D., Kern K.B., Patel N. (2016). Therapeutic Hypothermia and Stent Thrombosis: A Nationwide Analysis. JACC Cardiovasc. Interv..

[77] Farag M., Spinthakis N., Srinivasan M., Sullivan K., Wellsted D., Gorog D.A. (2018). Morphine Analgesia Pre-PPCI Is Associated with Prothrombotic State, Reduced Spontaneous Reperfusion and Greater Infarct Size. Thromb. Haemost..

[78] Hobl E.-L., Stimpfl T., Ebner J., Schoergenhofer C., Derhaschnig U., Sunder-Plassmann R., Jilma-Stohlawetz P., Mannhalter C., Posch M., Jilma B. (2014). Morphine decreases clopidogrel concentrations and effects: A randomized, double-blind, placebo-controlled trial. J. Am. Coll. Cardiol..

[79] Puymirat E., Lamhaut L., Bonnet N., Aissaoui N., Henry P., Cayla G., Cattan S., Steg G., Mock L., Ducrocq G. (2016). Correlates of pre-hospital morphine use in ST-elevation myocardial infarction patients and its association with in-hospital outcomes and long-term mortality: The FAST-MI (French Registry of Acute ST-elevation and non-ST-elevation Myocardial Infarction) programme. Eur. Heart J..

[80] Furtado R.H.M., Nicolau J.C., Guo J., Im K., White J.A., Sabatine M.S., Newby L.K., Giugliano R.P. (2020). Morphine and Cardiovascular Outcomes Among Patients With Non-ST-Segment Elevation Acute Coronary Syndromes Undergoing Coronary Angiography. J. Am. Coll. Cardiol..

[81] Iqbal J., Sumaya W., Tatman V., Parviz Y., Morton A.C., Grech E.D., Campbell S., Storey R.F., Gunn J. (2013). Incidence and predictors of stent thrombosis: A single-centre study of 5,833 consecutive patients undergoing coronary artery stenting. EuroIntervention.

[82] Batchelor R., Dinh D., Brennan A., Lefkovits J., Reid C., Duffy S.J., Cox N., Liew D., Stub D., VCOR Investigators (2020). Incidence, Predictors and Clinical Outcomes of Stent Thrombosis Following Percutaneous Coronary Intervention in Contemporary Practice. Heart Lung Circ..

[83] Champigneulle B., Haruel P.A., Pirracchio R., Dumas F., Geri G., Arnaout M., Paul M., Pène F., Mira J.P., Bougouin W. (2018). Major traumatic complications after out-of-hospital cardiac arrest: Insights from the Parisian registry. Resuscitation.

[84] Danial P., Hajage D., Nguyen L.S., Mastroianni C., Demondion P., Schmidt M., Bouglé A., Amour J., Leprince P., Combes A. (2018). Percutaneous versus surgical femoro-femoral veno-arterial ECMO: A propensity score matched study. Intensive Care Med..

[85] Pancholy S.B., Palamaner Subash Shantha G., Romagnoli E., Kedev S., Bernat I., Rao S.V., Jolly S., Bertrand O.F., Patel T.M. (2015). Impact of access site choice on outcomes of patients with cardiogenic shock undergoing percutaneous coronary intervention: A systematic review and meta-analysis. Am. Heart J..

[86] Gorog D.A., Price S., Sibbing D., Baumbach A., Capodanno D., Gigante B., Halvorsen S., Huber K., Lettino M., Leonardi S. (2021). Antithrombotic therapy in patients with acute coronary syndrome complicated by cardiogenic shock or out-of-hospital cardiac arrest: A joint position paper from the European Society of Cardiology (ESC) Working Group on Thrombosis, in association with the Acute Cardiovascular Care Association (ACCA) and European Association of Percutaneous Cardiovascular Interventions (EAPCI). Eur. Heart J. Cardiovasc. Pharmacother..

